# The Influence of ESG Ratings On Idiosyncratic Stock Risk: The Unrated, the Good, the Bad, and the Sinners

**DOI:** 10.1007/s41471-023-00155-1

**Published:** 2023-02-21

**Authors:** Matthias Horn

**Affiliations:** Department of Finance, Bamberg University, Kaerntenstraße 7, 96045 Bamberg, Germany

**Keywords:** ESG Rating, Idiosyncratic Risk, ESG Investments, Sin Stocks, CSR, Sustainable Investments, G11, G12, G24

## Abstract

This study analyzes whether stocks of companies with environmental social governance (ESG) rating show lower idiosyncratic risk. The main analysis covers 898,757 company-month observations of US stocks in the period from 1991 to 2018 and controls for stocks’ exposure to liquidity, mispricing, innovations in volatility risk, investor sentiment, and analysts’ forecast divergence. The main finding is that the receipt of an ESG rating decreases idiosyncratic stock risk. The effect is stronger for stocks that receive a higher ESG rating. Nevertheless, even when companies receive a lower ESG rating, they show significantly lower idiosyncratic risk than stocks without an ESG rating. Furthermore, stocks subject to a negative screen show lower idiosyncratic risk during recessions than comparable stocks with an ESG rating but without a negative screen. The results support the notion that the receipt of an ESG rating decreases uncertainty regarding future stock risk and return and show that ESG ratings and negative screens individually influence stock risk and, therefore, should be considered separately.

## Introduction

Risks associated with environmental, social, and governance (ESG) issues are idiosyncratic company risks. According to corner stone theories of the neoclassical finance paradigm, including the Capital Asset Pricing Model by Sharpe (1964), Lintner (1965), and Mossin (1966), expected stock returns do not depend on idiosyncratic company risk. In contrast, more recent empirical studies show that stocks with higher idiosyncratic risk, on average, exhibit significantly lower returns than stocks with lower idiosyncratic risk (see Ang et al. 2006, 2009; Stambaugh et al. 2015; Oehler and Schneider 2022). In consequence, investors only holding a limited number of stocks with high idiosyncratic risk may significantly harm their investment performance (see Levy, 1978; Adler and Kritzman 2008).

The purpose of ESG ratings is to measure the unmanaged ESG risk of a company. Ideally, ESG ratings decrease information asymmetries for the stakeholders of the company. However, the measurement of (unmanaged) ESG risk is very complex and there is considerable disagreement among ESG rating providers and different stakeholder groups of rated companies about the “correct” measurement approach. Nevertheless, ESG ratings significantly influence investment flows (see Benson and Humphrey 2008; Bialkowski and Starks 2016; Hartzmark and Sussman 2019; Latino et al. 2021). Hence, it is clear that investors actually consider ESG ratings in their investment decisions. The reason(s) to consider ESG ratings might differ among different types of investors. Some investors may regard the employed ESG rating as appropriate to assess ESG risk and use the rating in their stock selection process (see Renneboog et al. 2008; Oehler 2013; Riedl and Smeets 2017). Some institutional investors, such as ESG mutual funds, may commit themselves to only invest in stocks that receive an ESG rating (or stocks that are listed in an ESG index, which requires an ESG rating).

Importantly and somewhat surprising, though, many of the companies listed on stock exchanges do not receive an ESG rating, yet.^1^ This can have negative effects for the unrated companies and their shareholders, since the mere absence of an ESG rating can be a source of idiosyncratic risk. The reason for the existence of ESG ratings is that many investors are unable to assess the ESG risk of a company on their own, i.e., these investors are uncertain about the actual risk the company faces. Since uncertainty regarding a company’s risk is not covered by standard systematic risk measures (Anderson et al. 2009; Bali and Zhou 2016; Brenner and Izhakian 2018), it contributes to idiosyncratic stock risk. A decrease of information asymmetries reduces uncertainty and, therefore, idiosyncratic stock risk. Furthermore, some institutional investors cannot invest in stocks of unrated companies. However, institutional investors are considered to act rationally and help to price stocks efficiently (Boehmer and Kelley 2009), i.e., a lack of institutional investors increases idiosyncratic stock risk (Duppati et al. 2022).

Yet, the question whether the receipt of an ESG rating decreases idiosyncratic stock risk is unanswered in the literature. The present study answers this question of fundamental practical importance for investors considering an ESG investment^2^ approach and for companies deliberating on their ESG disclosure policy. The analysis spans the period from 1991 to 2018 and covers a survivorship bias-free sample of the stocks listed in the MSCI North America All Cap index. I apply ESG ratings from MSCI/KLD Stats and multiple widely recognized monthly factor and index data to control for market-wide liquidity (see Pástor and Stambaugh 2003), mispricing (see Stambaugh and Yuan 2017), investor sentiment and NBER recessions (see Baker and Wurgler 2006), and innovations in volatility risk (see Ang et al. 2006). ESG ratings are also provided for stocks of companies subject to a negative screen, i.e., companies associated with unethical products or “sin”, “vice”, or “controversial” industries. I control for negative screens in robustness checks due to their ambiguous standing in ESG approaches.

The contribution of this paper is fourfold. First, idiosyncratic stock risk decreases after the receipt of an ESG rating. Second, I provide empirical evidence that stocks of companies with an ESG rating show lower idiosyncratic risk than stocks of companies without an ESG rating—even when stocks receive a low ESG rating. Third, by showing that stocks subject to a negative screen show lower idiosyncratic risk during recessions than comparable stocks with an ESG rating but without a negative screen, this study shows that ESG ratings and negative screens have an individual influence on stock risk. This finding complements the notion of Zerbib (2020) that a company’s ESG rating and negative screen individually influence stock returns. Hence, ESG ratings and negative screens should be considered separately. Fourth, I show that the previously found relation between ESG ratings and idiosyncratic stock risk is not driven by stocks’ exposure to liquidity risk, mispricing, innovations in volatility risk, investor sentiment, analysts’ forecast divergence and coverage, and firms’ age, size, and industry and also holds for stocks with negative screens. Hence, I enhance the robustness of the finding that companies with higher ESG ratings show lower idiosyncratic stock risk (see e.g., Monti et al. 2019). These findings are also relevant for investors and stakeholders of non-American firms, particularly since a lot of non-American firms have not received an ESG rating, yet.

The remainder is structured as follows. With a focus on the ESG ratings provided by MSCI/KLD, the next section describes conceptual foundations, the rating process, and impacts of ESG ratings. Sect. 3 presents data sources and the methodology. Sect. 4 covers the main results. Robustness checks are provided in Sect. 5. Sect. 6 discusses the findings. Finally, Sect. 7 concludes.

## Conceptual Foundations, Rating Process, and Impacts of ESG Ratings

I focus on US stocks and respective MSCI/KLD ESG ratings (please note that these ratings are not identical with the MSCI ESG ratings)^3^. The advantage of this focus is that these ratings were provided several years before other rating providers entered the market.^4^ Therefore, it is very likely that the MSCI/KLD ESG rating is the first ESG rating a US company received, i.e., the results of this study are not biased by the earlier receipt of an ESG rating from a further rating provider. Furthermore, MSCI/KLD ESG ratings are considered the most comprehensive and widely-used data source for ratings in ESG research (Bouslah et al. 2013).

Like the approaches of most ESG ratings, MSCI/KLD ESG ratings offset companies’ strengths/opportunities against concerns/risks to measure the unmanaged ESG risk of the rated company. MSCI/KLD ESG ratings contain seven categories: *Community, Corporate Governance, Diversity, Employee Relations, Environment, Human Rights*, and* Product*. For each category, positive and negative performance indicators are provided to highlight strengths/opportunities and concerns/risks, respectively. The performance indicators are provided as binary values. Hence, the ESG ratings may rather be considered ESG performance scores than actual ratings. In addition, MSCI/KLD ratings include *Controversial Business Involvement Indicators*. I refer to these indicators as negative screens as they cover business involvement with alcohol, firearms, gambling, military, nuclear power, and tobacco (e.g., production or distribution of these products, being owner or supplier of a company involved with these products etc.; certain thresholds of involvement apply). Controversial business involvement may be correlated but is not identical to the industry sector of a company. Consider, e.g., the utility sector. Some producers of electric power may solely use renewable energy sources while other producers run nuclear power plants.

Similar to other rating providers, research analysts from MSCI/KLD collect data from many publicly available sources (e.g., companies’ sustainability reports, more than 1600 media sources, NGOs etc.). In addition, the rated companies are invited to review the data and the ESG report created by MSCI/KLD, i.e., companies can revise aspects and/or add private information. These revisions may cause changes of historical ESG ratings (Berg et al. 2021; Drempetic et al. 2021). Taking into account the latter ESG rating changes and the dispersion of the ratings of different ESG rating providers (Berg et al. 2022), it is clear that a single ESG rating is not a silver bullet to determine the ESG risk of a company. Nevertheless, ESG ratings should reduce information asymmetries and increase transparency regarding ESG issues. Since higher ESG transparency improves firm value by decreasing reputational risk, information asymmetries, agency costs, capital constraints, and ultimately, capital costs (Cheng et al. 2014; Erragragui 2018; Ng and Rezae 2015; Yu et al. 2018; Ghoul et al. 2011), companies should benefit from the receipt of an ESG rating—particularly from the first ESG rating and even when the disclosed information displays weak ESG performance (Eliwa et al. 2021). Not having an ESG rating can be interpreted as very low ESG transparency and, therefore, negatively affects firm value (Wong et al. 2021), i.e., is an idiosyncratic risk.

MSCI/KLD selects the companies to be rated by index membership and market capitalization. Over time, the ESG ratings usually have been provided for those stocks listed in the MSCI KLD 400 Social Index, S&P 500, MSCI USA Index, MSCI USA IMI Index, as well as the 1000 largest US companies. This selection approach of MSCI/KLD is an advantage for my empirical analysis. Every time when MSCI/KLD extends its coverage with a new index (e.g., the MSCI USA IMI Index in the year 2003), the setting is like a natural experiment in which hundreds of companies receive their first ESG rating while other company and stock characteristics (e.g., membership in a stock index, business model, financials) stay stable.^5^ It is worth mentioning that other ESG rating providers have different selection approaches that are not tied to stock index membership. Many of these other rating providers require the companies to be rated to collaborate, e.g., companies must fill out questionnaires or participate in interviews. Hence, some companies do not receive an ESG rating from the latter providers because they avoid or fail to collaborate.

Clearly, not all investors have direct access to ESG ratings. However, even these investors can identify the stocks with the highest ESG ratings, e.g., because of listings in sustainability indices or investments of ESG mutual funds. Hence, it is not surprising that ESG ratings significantly influence investment flows (see Benson and Humphrey 2008; Bialkowski and Starks 2016; Hartzmark and Sussman 2019; Latino et al. 2021). A key idea of this paper is that the first ESG rating should have the largest influence. As previous studies only consider the subsample of stocks corresponding to companies with an ESG rating, while ignoring the remaining stocks without an ESG rating, there is a gap in the literature on the relation between ESG ratings and idiosyncratic stock risk. This gap is of high relevance since many stocks around the globe do not receive an ESG rating, yet.

Previous studies as well as the present study are based on two assumptions. First, that stock markets are information-efficient when it comes to pricing in the available ESG ratings and, second, that the ESG ratings properly proxy the ESG risk exposure of the rated companies. Particularly the second assumption is hard to prove. Mild support of the robustness of these assumptions is that earnings forecasts for stocks with higher ESG ratings are more precise (Becchetti et al. 2013) and that companies with lower ESG ratings are more likely to become bankrupt (Cooper and Uzun 2019). Stronger support is found by Serafeim and Yoon (2021), showing that ESG ratings predict future company-specific ESG news and—with some constraints—proxy for market expectations of future ESG news. Accordingly, studies generally hypothesize (see e.g., Bouslah et al. 2018; for a theoretical framework; see also Friede et al. 2015) and followingly find that stocks of companies with higher ESG ratings show lower idiosyncratic risk than stocks of companies with lower ESG ratings (Mishra and Modi (2013), Becchetti et al. (2015), Sassen et al. (2016), Bouslah et al. (2013, 2018), Dunn et al. (2018), Giese et al. (2019), and Monti et al. (2019)).^6^

The question whether the mere receipt of an ESG rating decreases idiosyncratic stock risk is of fundamental practical importance for investors and companies. Investors following ESG investment approaches may be substantially exposed to idiosyncratic risk and thereby suffer from weak investment performance, even with portfolios of several hundred stocks (see Barnett and Salomon 2006; Geczy et al. 2003; Statman 2000; Levy 1978; Adler and Kritzman 2008). The reason is that ESG investment approaches limit the number of investable stocks by prohibiting investments in certain “sin”, “vice”, or “controversial” industries or companies associated with unethical products (usage of negative screens), or only allow investments in the most sustainable companies of a peer group (best-in-class approach) (see Fabozzi et al. 2008; Oehler et al. 2018; Zerbib 2020). If the receipt of an ESG rating mitigates idiosyncratic risk, it is less likely that ESG investors experience underperformance. Furthermore, companies have an incentive to get rated when ESG ratings reduce idiosyncratic stock risk and, thus, capital costs.

## Data and Methodology

The analysis employs MSCI/KLD ESG ratings for US stocks on a yearly basis from 1991 to 2018.^7^ To check the obtained results for robustness as well as to derive potential implications, I also include Canadian stocks into the analysis. For Canadian stocks, the ESG ratings are available from 2013 to 2018.

I use daily total return and stock price data from Thomson Reuters Datastream from January 1991 to the end of 2018. Stocks with a price lower than five US Dollars at the beginning of a month are excluded for that month (see Pástor and Stambaugh 2003; Stambaugh et al. 2015).^8^ The idiosyncratic stock risk per month is based on daily returns and the Carhart (1997) four-factor model, which is defined as:

1 $$R_{it}-R_{Ft}=\beta _{1i}*R_{Mt}+\beta _{2i}*\mathrm{SMB}_{t}+\beta _{3i}*\mathrm{HML}_{t}+\beta _{4i}*\mathrm{WML}_{t}+\alpha _{i}+\varepsilon _{i{,}t}$$

where *R*_*it*_ is the return of stock *i* and *R*_*Ft*_ is the risk-free return on day *t*. *R*_*Mt*_, *SMB*_*t*_, *HML*_*t*_, are the three factors defined by Fama and French (1992). *WML*_*t*_ is the momentum factor introduced by Carhart (1997). The betas in Eq. 1 are calculated by running Eq. 1 every month for each individual stock *i*. *ε*_*i*,*t*_ is the residual per day *t*. Idiosyncratic risk of stock *i* measured as daily idiosyncratic volatility per month *m* (IVOL_i,m_; in the remainder of the paper referred to as IVOL for better readability) is defined as $\sqrt{var\left(\varepsilon _{i{,}t}\right)}$ for all days *t* of month *m* (for this approach, see Bouslah et al. 2018).^9^ All results for IVOL are presented in percent. As robustness check, IVOL_i,m_ is also computed with the Fama and French (2015) five factor model. The daily North American factors for the four- and five-factor model are from Kenneth French’s homepage.^10^ As further robustness check, IVOL is trimmed and winsorized to minimize the influence of outliers. I do not winsorize or trim the values of IVOL in the main analysis since the occurrence of ESG risks might cause severe IVOL. Winsorizing or trimming these effects away might lead to an underestimation of the relation between ESG ratings and IVOL.

T‑tests, an event study approach, and panel regressions with random effects and robust standard errors clustered by company are provided to analyze differences in IVOL of stocks with and without ESG rating. The presence of an ESG rating for a company is indicated with a dummy variable (*HasESGRating*). Since companies with a higher ESG rating show lower idiosyncratic stock risk, I include the ESG rating and employ an interacted variable containing the ESG rating dummy and the ESG rating (HasESGRating*ESGRating). This interacted variable shows whether companies that receive a higher ESG rating benefit more from the receipt of the rating than companies with a lower ESG rating. I compute the ESG rating with the approach used by Lins et al. (2017), however, including all seven categories instead of only applying five. For each category I compute a score which is the sum of strength divided by the maximum number of strengths possible for that category in that year minus the sum of concerns divided by the maximum number of concerns possible. The overall ESG rating (*ESGRating*) is the sum of the seven category scores and may range between +7 and −7. As negative screens are not inclusive in the ESG rating, companies subject to a negative screen can still reach the best possible ESG rating of +7.

In the regression analyses, the influence of negative screens on idiosyncratic risk is controlled for by adding a dummy variable, which equals one if a company is subject to any negative screen (*NegativeScreen*). The dummy is included since firms subject to a negative screen must pay higher costs of equity (Chava 2014; Hong and Kacperczyk 2009; Ghoul et al. 2011; Killins et al. 2020), higher costs for loans (Chava 2014; Goss and Roberts 2011; Kim et al. 2014; Nandy and Lodh 2012), and face capital constraints (see, e.g., initiatives like Net Zero Asset Managers). However, in market downturns when idiosyncratic risk rises with market risk (Bartram et al. 2016), stocks of sin companies may profit from their defensive nature (Richey 2020).

As the ESG ratings are provided on a yearly basis, there are no changes over the twelve monthly observations of each year, causing the R^2^ of the regression analyses to remain rather low. Nevertheless, I choose the monthly panel regression approach since the regressions apply multiple firm characteristics as well as factors from different models to capture systematic time-series variations in realized returns (see Stambaugh and Yuan 2017) that may appear on monthly time horizons and only temporarily influence idiosyncratic stock risk. The applied factors control for liquidity risk (*InnovLiq*; see Pástor and Stambaugh 2003; also referred to as the non-traded liquidity factor to capture innovations in market liquidity and to estimate an asset’s liquidity risk, see Pástor and Stambaugh 2019)^11^, mispricing (*SMB_Mispricing, MGMT_Mispricing, PERF_Mispricing*; see Stambaugh and Yuan 2017)^12^, and investor sentiment^13^ (*Sentiment*; see Baker and Wurgler 2006; Stambaugh et al. 2012). Furthermore, due to their impact on stock returns and idiosyncratic risk, analyst forecast divergences (*Deviation_Analysts*; see Boehme et al. 2009; Diether et al. 2002) and analyst coverage (*Number_Analysts*; see Chichernea et al. 2015; Cao and Han 2016)^14^, industry sectors^15^ (*Industry_Sectors*; see Moskowitz and Grinblatt 1999), NBER recessions^16^ (*USREC*; see Bozhkov et al. 2020), innovations in volatility risk proxied as changes in the S&P 500 VIX (∆*VIX*; see Ang et al. 2006), market capitalization (*Size;* the log market capitalization in million US Dollars), and firm age (*Age*; log age defined as the number of years since the first date of trading of the stock; see Pástor and Veronesi 2003; Ferreira and Laux 2007; Cao et al. 2008)^17^ are also included. Some company-month observations suffer from missing data. Data for *SMB_Mispricing, MGMT_Mispricing, PERF_Mispricing* is only available until the end of the year 2016 and, therefore, missing for 76,305 observations in the years 2017 and 2018. *Deviation_Analysts* is only available for 529,657 observations, *Number_Analysts* for 532,772 observations, *Age* for 835,662 observations, *Size* for 898,367 observations and *Industry_Sectors* for 807,195 observations.

Since endogeneity effects may influence the results of the panel regressions, a matching and difference-in-differences approach for causal analysis with varying treatment time and duration is employed as robustness check (see Dettmann et al. 2020). Due to the weaknesses associated with propensity score matching (King and Nielsen 2019), the approach is based on coarsened exact matching (see Blackwell et al. 2009). As further robustness check, I split the dataset in observations before and after the end of the financial crisis and rerun the regression analysis (see Lins et al. 2017; and SIF 2018).

**Fig. 1:**
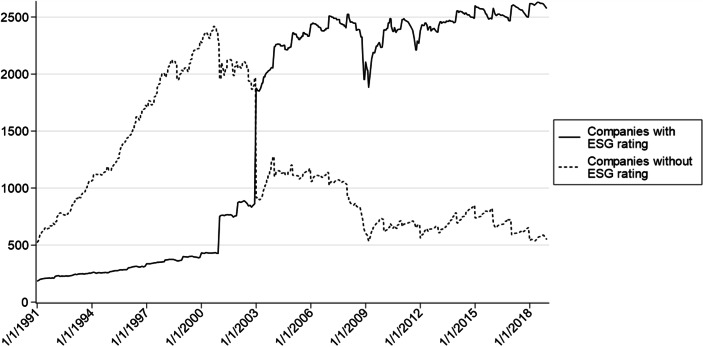
Number of companies per month in the period from January 1991 to December 2018

Figure 1 shows a plot of the number of US companies per month included in the analysis subdivided in companies with and without an ESG rating. Please note that observations of stocks with a price lower than five US Dollars are not included in the plot and the analysis. Since stock prices considerably plunged during the financial crisis in the year 2008, the five US Dollars hurdle serves as explanation for the decrease in the number of stocks with an ESG rating as well as the number of stocks without an ESG rating in this period. In January 1991, 707 stocks, of which 184 received an ESG rating, are considered. In the years 2001 and 2003, MSCI significantly expanded the rating coverage. Since then, the number of stocks with an ESG rating increases while the number of unrated stocks decreases. At the end of the year 2018, the sample covers 2576 stocks with an ESG rating and 552 stocks without an ESG rating.

## Main Results

The main results are based on the sample of US stocks, which covers 898,757 company-month observations. Table 1 provides descriptive statistics of these companies’ ESG ratings, IVOL, Size, and Age.

**Table 1:** Descriptive statistics of the companies’ ESG ratings and IVOL_i,m_ in the period from January 1999 to December 2018

	Full sample	Observations with ESG rating			Observations without ESG rating
		All	Without negative screen	With negative screen	
*ESGRating*					
Mean	–	0.07	0.06	0.11	–
Median	–	0.00	0.00	0.00	–
Std. dev	–	0.81	0.79	1.01	–
N	–	516,569	467,771	48,798	382,188
*IVOL*					
Mean	1.99	1.63	1.65	1.40	2.47
Median	1.53	1.32	1.34	1.16	1.93
Std. dev	1.92	1.20	1.22	0.95	2.51
N	898,757	516,569	467,771	48,798	382,188
*Size*					
Mean	6.56	7.37	7.28	8.17	5.47
Median	6.48	7.23	7.15	8.18	5.29
Std. dev	1.86	1.61	1.57	1.76	1.61
N	898,367	516,563	467,765	48,798	381,804
*Age*					
Mean	2.38	2.60	2.57	2.92	2.05
Median	2.56	2.83	2.77	3.14	2.20
Std. dev	0.99	0.91	0.92	0.84	1.01
N	835,662	495,355	447,965	47,390	340,307
*Number_Analysts*					
Mean	7.18	7.87	7.67	9.93	3.83
Median	5	6	6	9	3
Std. dev	5.98	6.15	6.05	6.75	3.48
N	532,772	441,434	402,108	39,326	91,338

ESG ratings are available for 516,569 observations in an unbalanced panel. The ESG ratings have a mean (median) value of 0.07 (0.00) and range between −3.25 and 5.9. Companies subject to a negative screen on average have a 0.05 points higher ESG score than companies not subject to a negative screen. This difference is significant at the one per mill level and again underlines the importance to differentiate between negative screens and the remaining ESG aspects (see Zerbib 2020). The mean IVOL of the full sample is 1.99% with a standard deviation of 1.92%. When the full sample is split up in stocks of companies with and without ESG rating, the mean IVOL of stocks with ESG rating is 0.85 percentage points lower than the IVOL of stocks with no ESG rating. Companies with ESG rating are larger, older, and covered by more analysts than stocks of unrated companies with a statistical significance at the one per mill level. Among the rated stocks, those without a negative screen are on average smaller, younger, and covered by fewer analysts with a statistical significance at the one per mill level.

**Fig. 2:**
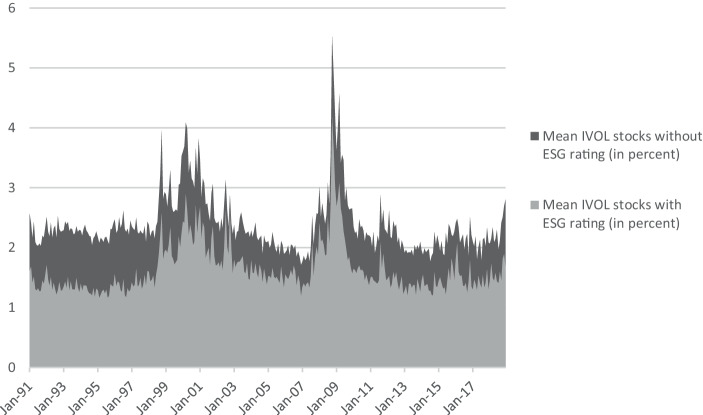
Mean IVOL_i,m_ per month in percent for US stocks without and with ESG rating in the period from January 1991 to December 2018

Figure 2 shows the difference in IVOL of stocks with and without ESG rating per month in the period from January 1991 to December 2018. Stocks with ESG rating on average show a lower IVOL in every month of the 28-year long observation period. T‑tests with Welch’s (1947) formula show that the IVOL differences are significant at the one per mill level in each month.

The t‑tests only show that the IVOL of stocks of companies that already received an ESG rating is lower. But of particular interest is the question whether receiving an ESG rating by itself leads to lower idiosyncratic stock risk or whether rating providers are biased in their selection and, thus, only rate companies with already lower idiosyncratic risk (for a discussion of the comparable reverse causality issue regarding ESG ratings, see e.g., Krueger 2015). This question is analyzed with an event study approach. Stocks of 1840 companies received an ESG rating for the first time in the years 1993–2014 and have been traded on the market for at least two years before and at least four years after the receipt of their first ESG rating. The IVOL of these stocks is compared to the IVOL of all other stocks (i.e., a sample of stocks with and without an ESG rating). The month of the receipt of the ESG rating is set as *t* = 0.

**Fig. 3:**
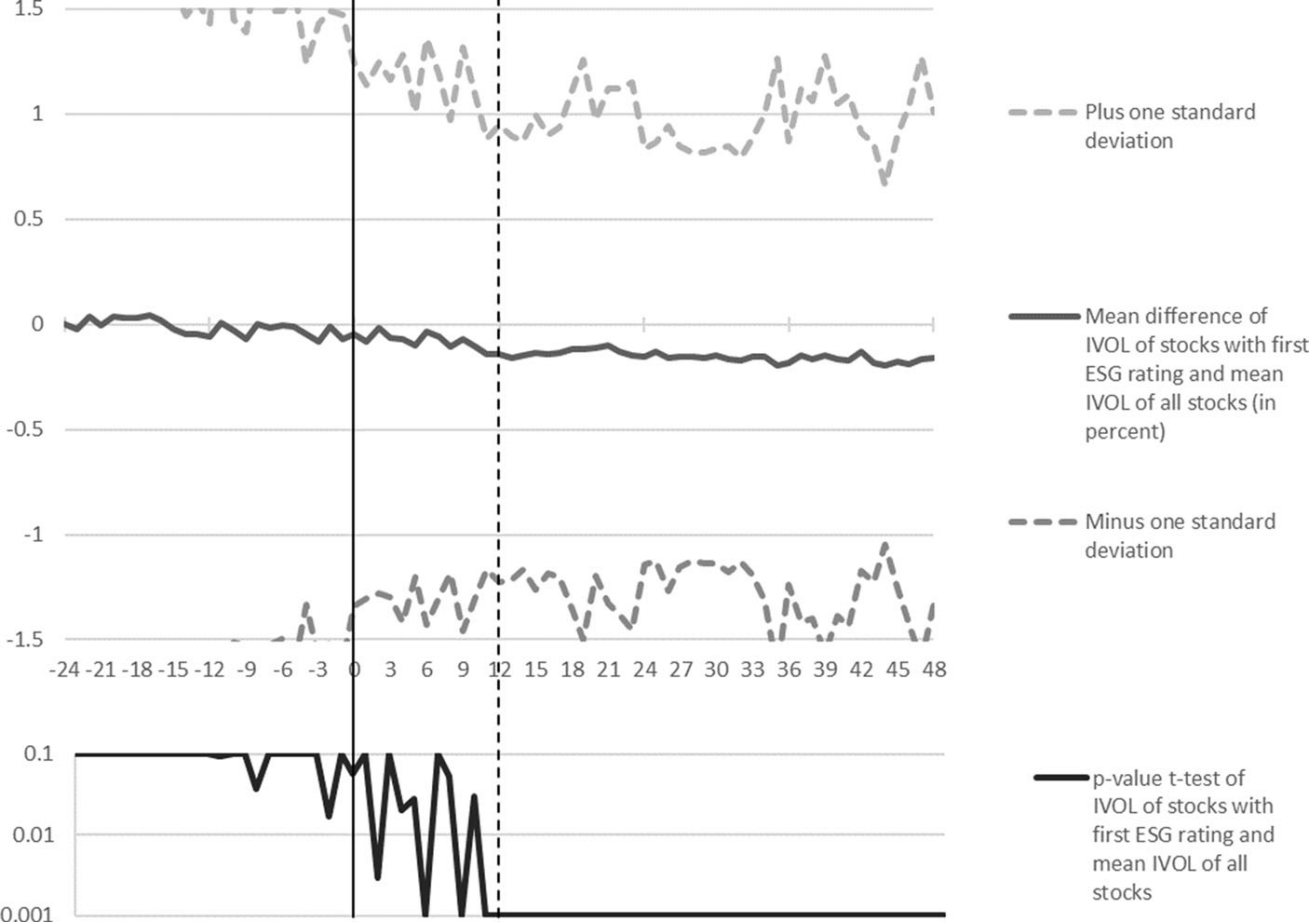
Mean difference of IVOL of stocks receiving their first ESG rating and IVOL of all stocks in the period spanning 24 months before and 48 months after the rating receipt

The upper part of Fig. 3 shows the mean difference in IVOL of stocks around the receipt of their first ESG rating and the IVOL of all stocks. Please note that due to the construction of the employed MSCI/KLD dataset, market participants might actually have got the information regarding the new rating up to twelve months later than *t* = 0. In the months −24 to 0 the mean IVOL of stocks that will receive an ESG rating in *t* = 0 is not different from the mean IVOL of all stocks at statistically significant levels (see the respective *p*-values of t‑test per month in the lower part of Fig. 3). In the months 0 to twelve, when the information about the received rating spreads among market participants, the mean IVOL of newly rated stocks is significantly decreasing compared to the IVOL of all stocks. In months twelve to 48, the mean IVOL of the stocks that received an ESG rating is on average 15 percentage points lower than the mean IVOL of all stocks. The latter difference is statistically significant at the one per mill level in each month of the period from months twelve to 48. Furthermore, the distribution of stocks’ IVOL clearly becomes narrower when the respective companies received an ESG rating. This is in line with the conjecture that the receipt of an ESG rating decreases uncertainty regarding future risk and return. The mean standard deviation of the difference between the IVOL of newly rated stocks and the mean IVOL of all stocks is 1.66% in months −24 to −1, 1.22% in months 0 to eleven, and 1.13 in months twelve to 48. Hence, results of the event study approach show that the receipt of an ESG rating does not only decrease a stock’s mean idiosyncratic risk, but also narrows the width of a stock’s IVOL distribution.

To analyze the causal relationship between the receipt of an ESG rating and idiosyncratic stock risk in further detail, I apply stepwise panel regressions. The regression results presented in Table 2 support the results of the t‑tests and the event study approach; ESG-rated stocks show lower IVOL than stocks of companies without a rating. The coefficient of the respective dummy variable (*HasESGRating*) is significant at the one per mill level in all model specifications. In addition, the results support the previously found negative relation between a company’s ESG rating and its IVOL (see Mishra and Modi 2013; Sassen et al. 2016; Bouslah et al. 2018; Dunn et al. 2018; and Giese et al. 2019). Hence, companies that receive a high ESG rating benefit even more from the receipt of the ESG rating. But even the IVOL of companies that receive a low ESG rating decreases: The coefficient of HasESGRating*ESGRating times the minimum ESG rating in the sample (−3.25) is smaller in magnitude than the coefficient of *HasESGRating* in all models of Table 2 (e.g. −0.11 * −3.25 = 0.36 vs. −0.75 in model (2)). In recessions, the extenuating effect of an ESG rating on IVOL is smaller. As expected, stocks subject to a negative screen show lower IVOL in recessions, i.e., stocks of sin companies profit from their defensive nature (see Richey 2020).^18^ However, in the remaining time, sin stocks show a higher IVOL than stocks not subject to a negative screen. Hence, stocks subject to a negative screen show a higher IVOL in total, when adjusted for their ESG rating. Results of the full regression model show that the risk decreasing effect of an ESG rating cannot be explained by exposure to liquidity risk, mispricing, innovations in volatility risk, investor sentiment, analysts’ forecast divergence and coverage, and firms’ age, size, and industry.

**Table 2:** Panel regressions on IVOL_i,m_ in percent for US stocks without and with ESG rating

	(1)	(2)	(3)	(4)	(5)	(6)	(7)	(8)	(9)	(10)
HasESGRating	−0.75**** (0.02)	−0.75**** (0.02)	−0.79**** (0.02)	−0.74**** (0.02)	−0.74**** (0.02)	−0.75**** (0.02)	−0.72**** (0.02)	−0.91**** (0.02)	−0.18**** (0.02)	−0.62**** (0.03)
HasESGRating*ESGRating	–	−0.11**** (0.01)	−0.07**** (0.01)	−0.10**** (0.01)	−0.12**** (0.01)	−0.11**** (0.01)	−0.11**** (0.01)	−0.09**** (0.01)	0.00 (0.00)	−0.01** (0.01)
NegativeScreen	–	–	0.08*** (0.03)	–	–	–	–	–	–	0.07* (0.03)
HasESGRating*USREC	–	–	0.40**** (0.03)	–	–	–	–	–	–	0.57**** (0.03)
NegativeScreen*USREC	–	–	−0.21**** (0.04)	–	–	–	–	–	–	−0.15* (0.04)
USREC	–	–	0.54**** (0.03)	–	–	–	–	–	–	0.25**** (0.03)
InnovLiq	–	–	–	−1.51**** (0.03)	–	–	–	–	–	−0.62**** (0.04)
SMB_Mispricing	–	–	–	–	0.57**** (0.08)	–	–	–	–	−0.25**** (0.07)
MGMT_Mispricing	–	–	–	–	1.76**** (0.08)	–	–	–	–	1.76**** (0.08)
PERF_Mispricing	–	–	–	–	0.74**** (0.04)	–	–	–	–	−0.28**** (0.04)
∆VIX	–	–	–	–	–	0.02**** (0.00)	–	–	–	0.02**** (0.00)
Sentiment	–	–	–	–	–	–	0.06**** (0.01)	–	–	−0.01 (0.01)
Deviation_Analysts(x 10^−6^)	–	–	–	–	–	–	–	3.83* (1.76)	–	1.42 (2.102)
Number_Analysts	–	–	–	–	–	–	–	−0.03**** (0.00)	–	0.01**** (0.00)
Size	–	–	–	–	–	–	–	–	−0.37**** (0.02)	−0.31**** (0.01)
Age	–	–	–	–	–	–	–	–	−0.18**** (0.01)	−0.24**** (0.01)
Industry_Sectors	No	No	No	No	No	No	No	No	Yes	Yes
$\beta _{0}$	2.64**** (0.02)	2.64**** (0.02)	2.60**** (0.02)	2.64**** (0.02)	2.61**** (0.02)	2.62**** (0.02)	2.61**** (0.02)	2.89**** (0.02)	5.06**** (0.11)	4.98**** (0.10)
R^2^	0.05	0.05	0.06	0.05	0.05	0.05	0.05	0.07	0.19	0.24
N	898,666	898,666	898,666	898,666	822,452	892,433	898,666	506,566	736,974	383,514

I provide coefficients, robust standard errors (in parentheses) clustered by company, and R^2^ for random-effects panel regression analysis with the model $\mathrm{IVOL}_{i{,}m}=\beta _{1i}*\text{HasESGRating}_{i{,}m}+\beta _{2i}*\text{HasESGRating}_{i{,}m}*\text{ESGRating}_{i{,}m}+\beta _{3i}*\text{NegativeScreen}_{i{,}m}+\beta _{4i}*\text{HasESGRating}_{i{,}m}*\text{USREC}_{m}+\beta _{5i}*\text{NegativeScreen}_{i{,}m}*\text{USREC}_{m}+\beta _{6i}*\text{USREC}_{m}+{\sum }_{j=7}^{12}\beta _{j{,}i}*X_{j{,}m}+\beta _{13i}*\text{Deviation}\_ \text{Analysts}_{i{,}m}+\beta _{14i}*\text{Number}\_ \text{Analysts}_{i{,}m}+\beta _{15i}*\text{Size}_{i{,}m}+\beta _{16i}*\mathrm{Age}_{i{,}m}+\gamma *IS_{i}+\beta _{0{,}i}+u_{i{,}m}$, where *IVOL*_*i*,*m*_ is the idiosyncratic risk of stock i measured as daily idiosyncratic volatility per month *m* in percent in the period from January 1999 to December 2018, HasESGRating_*i*,*m*_ is a dummy variable indicating whether a stock *i* has an ESG rating in month *m*, ESGRating_*i*,*m*_ is the ESG rating of stock *i* in month *m*, *NegativeScreen*_*i*,*m*_ is a dummy variable indicating whether a stock *i* is subject to a negative screen in month *m*, *USREC*_*m*_ is a dummy variable indicating whether the United States have been in a recession in month *m*, *X*_7,*m*_, …, *X*_12,*m*_ are the factors InnovLiq, SMB_Mispricing, MGMT_Mispricing, PERF_Mispricing, ∆VIX, and Sentiment. $\text{Deviation}\_ \text{Analysts}_{i{,}m}$ is the standard deviation of analysts’ price forecasts for stock *i* in month *m*, $\text{Number}\_ \text{Analysts}_{i{,}m}$ is the number of analysts that provide price forecasts for stock *i* in month *m*, Size_*i*,*m*_ is the log market capitalization in million US Dollars of stock *i* in month *m*, Age_*i*,*m*_ is the log number of years since the first date of trading of stock *i* in month *m*, and *IS*_*i*_ is vector of nine dummy variables to reflect firms’ industrial sector

Please note that the factor data for SMB_Mispricing, MGMT_Mispricing, and PERF_Mispricing applied in models (5) and (10) is only available until the end of the year 2016

Hence, models (5) and (10) cover the period from January 1999 to December 2016

The symbols ****, ***, **, and * denote significance at the one per mill, five per mill, one percent, and five percent level, respectively

Coefficients with *p*-values ≥ 0.05 are not labeled as significant

Example: Regressing *IVOL*_*i*,*m*_ on the dummy variable HasESGRating_*i*,*m*_ (model (1)) yields a coefficient of −0.75 with a *p*-value < 0.001 for this dummy variable

## Robustness Checks

To address the issue that outliers may drive the results, the values of IVOL are winsorized at the lowest (five percent) and highest (95%) percentile each month. The coefficients of *HasESGRating* as reported in Table 2 change by at most two percentage points for the one and 99% winsorization and by at most three percentage points for the five and 95% winsorization. The coefficients remain their statistical significance at the one per mill level. Trimming the values of IVOL_i,m_ at the lowest and highest percentile each month has the same effect on the regression results as the respective winsorization.

Instead of using the four-factor model of Carhart (1997), some investors may assess idiosyncratic risk rather by applying the more recent five-factor model of Fama and French (2015). Hence, I additionally compute IVOL_i,m_ (denoted as IVOL_i,m_^5F^) with this model.^19^ Compared to the previous analyses, the untabulated results for IVOL_i,m_^5F^ as dependent variable are almost identical^20^ and therefore robust with regard to the applied factor model.

As reported by Lins et al. (2017), stocks of companies with high ESG ratings earned a premium during the financial crisis. Thereafter, investments in ESG mutual funds soared (see SIF 2018), indicating that market participants’ investment approaches may have been subject to change, leading to an increased awareness—and, thus, an increased demand—with regard to ESG investment approaches^21^. Therefore, I split the dataset in observations before and after the end of the financial crisis and repeat the regression analyses for the latter subsample. Following NBER data on economic cycles, July 2009 is set as the end of the financial crisis (first month not marked as a recession since the beginning of the financial crisis). The respective results presented in Table 3 show that the IVOL of stocks with and without rating converges but is still significantly higher for stocks without an ESG rating. However, when the full model is employed, the difference between the IVOL of rated and unrated stocks vanishes. It is worth noticing that the latter observation may be driven by the exclusion 163,964 company-month observations that are omitted because of missing data on the independent variables.

**Table 3:** Panel regressions on IVOL_i,m_ per month for observations from July 2009 and later

	(1)	(2)	(3)
HasESGRating	−0.36**** (0.03)	−0.34**** (0.03)	0.00 (0.04)
HasESGRating*ESGRating	–	−0.05**** (0.01)	−0.00 (0.00)
NegativeScreen	–	−0.05 (0.02)	0.03 (0.02)
InnovLiq	–	–	−0.02 (0.06)
SMB_Mispricing	–	–	−0.65**** (0.09)
MGMT_Mispricing	–	–	1.93**** (0.13)
PERF_Mispricing	–	–	−0.17*** (0.06)
∆VIX	–	–	0.01**** (0.00)
Sentiment	–	–	−0.16**** (0.01)
Deviation_Analysts(x 10^−6^)	–	–	−4.01 (14.9)
Number_Analysts	–	–	0.02**** (0.00)
Size	–	–	−0.37**** (0.02)
Age	–	–	−0.03* (0.01)
Industry_Sectors	No	No	Yes
$\beta _{0}$	2.12**** (0.03)	2.11**** (0.03)	4.25**** (0.11)
R^2^	0.03	0.04	0.20
N	359,615	359,615	195,651

I provide coefficients, robust standard errors (in parentheses) clustered by company, and R^2^ for random-effects panel regression analysis with the model $\mathrm{IVOL}_{i{,}m}=\beta _{1i}*\text{HasESGRating}_{i{,}m}+\beta _{2i}*\text{HasESGRating}_{i{,}m}*\text{ESGRating}_{i{,}m}+\beta _{3i}*\text{NegativeScreen}_{i{,}m}+{\sum }_{j=4}^{9}\beta _{j{,}i}*X_{j{,}m}+\beta _{10i}*\text{Deviation}\_ \text{Analysts}_{i{,}m}+\beta _{11i}*\text{Number}\_ \text{Analysts}_{i{,}m}+\beta _{12i}*\text{Size}_{i{,}m}+\beta _{13i}*\mathrm{Age}_{i{,}m}+\gamma *IS_{i}+\beta _{0{,}i}+u_{i{,}m}$, where *IVOL*_*i*,*m*_ is the idiosyncratic risk of stock *i* measured as daily idiosyncratic volatility per month *m* in percent in the period from July 2009 to December 2018, HasESGRating_*i*,*m*_ is a dummy variable indicating whether a stock *i* has an ESG rating in month *m*, ESGRating_*i*,*m*_ is the ESG rating of stock *i* in month *m*, *NegativeScreen*_*i*,*m*_ is a dummy variable indicating whether a stock *i* is subject to a negative screen in month *m*, *X*_4,*m*_, …, *X*_9,*m*_ are the factors InnovLiq, SMB_Mispricing, MGMT_Mispricing, PERF_Mispricing, ∆VIX, and Sentiment. $\text{Deviation}\_ \text{Analysts}_{i{,}m}$ is the standard deviation of analysts’ price forecasts for stock *i* in month *m*, $\text{Number}\_ \text{Analysts}_{i{,}m}$ is the number of analysts that provide price forecasts for stock *i* in month *m*, Size_*i*,*m*_ is the log market capitalization in million US Dollars of stock *i* in month *m*, Age_*i*,*m*_ is the log number of years since the first date of trading of stock *i* in month *m*, and *IS*_*i*_ is vector of nine dummy variables to reflect firms’ industrial sector

Please notice that the factor data for SMB_Mispricing, MGMT_Mispricing, and PERF_Mispricing applied in model (3) is only available until the end of the year 2016

Hence, model (3) covers the period from July 2009 to December 2016

The symbols ****, ***, **, and * denote significance at the one per mill, five per mill, one percent, and five percent level, respectively

Coefficients with *p*-values ≥ 0.05 are not labeled as significant

Example: Regressing *IVOL*_*i*,*m*_ on the dummy variable HasESGRating_*i*,*m*_ (model (1)) yields a coefficient of −0.36 with a *p*-value < 0.001 for this dummy variable

Due to the significant increase of the universe of rated stocks in the year 2003, I split up the sample in observations before and after January 1st, 2003. The untabulated results for the latter subsample are very similar to those reported in Table 3 for the subsample covering the period after the financial crisis.^22^ In addition, I find further support that stocks subject to a negative screen showed statistically significant lower idiosyncratic risk during the financial crisis than comparable stocks with an ESG rating but without a negative screen. Results for observations before the year 2003 show a significant negative relation between *HasESGRating* and IVOL when the full regression model is applied.

To address endogeneity issues regarding the companies’ first receipt of an ESG rating, i.e., whether rating providers are biased in their selection and only rate companies with already lower idiosyncratic risk, I provide a matching and difference-in-differences approach for causal analysis with varying treatment time and duration (see Dettmann et al. 2020). The considered time window for the matching approach spans the years 2000 to 2018. The reasons for this choice are that MSCI/KLD significantly expanded the rating coverage in the years 2001 and 2003 by about 1750 companies and that the popularity of ESG investment approaches soared after the financial crisis. The matching of the stocks is based on two sets of matching criteria. The first set includes industry sector and firm size measured as market capitalization, due to their important role in the ESG rating process and their influence on ESG scores (see e.g., Monti et al. 2019; Drempetic et al. 2020). The second set covers stocks’ loadings on the five factors of the Fama and French (2015) model in month *t* to capture stocks’ exposure to systematic risk factors at the time just before an ESG rating has been assigned. Companies that receive an ESG rating in month *t* for the first time are matched with companies that do not receive an ESG rating in month *t* according to the matching criteria in month *t* − 1. The difference-in-differences approach compares the IVOL of the matched companies in month *t* − 1 with their IVOL in months *t* + 13, *t* + 24, and *t* + 36. The robustness of the conditional difference-in-differences is checked with a fixed effects panel regression to compute the total treatment effect for the treated companies within a two-way fixed effects model. Due to the significant role of the financial crisis, as before, the dataset is split in observations before and after the end of the financial crisis.^23^

Baker et al. (2022) show that the results of staggered difference-in-differences analyses can be biased. A major source of biased results is a violation of the parallel trends assumption, i.e., that the IVOL of the stocks in the treated group (stocks that received an ESG rating)—if they had not been treated—would have followed the same trend as the IVOL of the stocks that did not receive an ESG rating (the untreated). The plots in Fig. 2 show that the trends of the IVOL of stocks with and without ESG rating are highly correlated; the respective correlation coefficient is 0.91. Furthermore, the difference between the IVOL of stocks with and without ESG rating stays stable over time, i.e., the trends show parallelism (exceptions are times of crashes). In addition to this support for the assumption of parallel trends, Ryan et al. (2018) show that difference-in-differences analyses in combination with matching of observations show reasonable outcomes, even when the parallel trends assumption is violated. Hence, the difference-in-differences analysis should yield robust results.

The results of the difference-in-differences analysis are presented in Table 4 and 5. Unsurprisingly, not all companies that receive their first ESG rating can be matched with an unrated company. Depending on the analyzed time window and employed matching criteria, 63–96% of the newly rated companies can be matched. Results for stocks matched by market capitalization and industry sector are presented in Table 4. The IVOL of the stocks that received their first ESG rating on average decreased by −0.13 to −0.53 percentage points when compared to the IVOL in the month before the receipt of the first rating. This reduction in IVOL is 0.14 to 0.52 larger than the reduction comparable stocks without an ESG rating experienced. The conditional difference-in-differences is significant at the one per mill level before the financial crisis. Statistical significance after the financial crisis is lower, but still reaches the one percent level for the 36 months observation period. The total effect of the ESG rating receipt in a two-way fixed effects model with robust standard errors also is negative. However, only in the model covering observations before the financial crisis in a 24-month period, the respective coefficient is statistically significant at the one per mill level. Results for stocks matched by factor loadings according to the Fama and French (2015) five factor model are shown in Table 5. The results are similar to those based on the market capitalization and industry sector-matching. The only noteworthy exception is that the negative total effect of the ESG rating receipt on IVOL in the two-way fixed effects model is significant at the five percent level in the models covering observations after the financial crisis in a 24-month and 36-month period. Hence, the results of the difference-in-differences analysis are further support that the receipt of an ESG rating decreases idiosyncratic stock risk.

**Table 4:** Difference-in-differences approach applied on IVOL of stocks receiving their first ESG rating (i.e., the treatment) and IVOL of stocks without ESG rating (control group) based on CE Matching by market capitalization and industry sector

	(1)	(2)	(3)	(4)	(5)	(6)
*Time period*	Jan 1999–June 2009	Jan 1999–June 2009	Jan 1999–June 2009	July 2009–Dec 2018	July 2009–Dec 2018	July 2009–Dec 2018
*Mean difference treated*	−0.38	−0.41	−0.53	−0.13	−0.43	−0.49
*Mean difference controls*	−0.17	−0.27	−0.35	0.01	−0.05	0.03
*Conditional difference-in-differences (standard error of differences between treated and control group in parentheses)*	−0.21**** (0.03)	−0.14**** (0.04)	−0.18**** (0.04)	−0.14 (0.12)	−0.39* (0.15)	−0.52** (0.20)
*Total treatment effect for the treated within two-way fixed effects model (robust standard error in parentheses)*	−0.13 (0.10)	−0.38**** (0.11)	−0.14 (0.14)	−0.04 (0.22)	−0.36 (0.21)	−0.29 (0.24)
*N treated matched*	1585	1387	1248	540	406	305
*N controls matched*	565	464	385	584	478	373
*N treated unmatched*	75	95	87	17	15	12
*Matching variables included:*						
Market capitalization	Automatic	Automatic	Automatic	Automatic	Automatic	Automatic
Industry sector	Cutpoints	Cutpoints	Cutpoints	Cutpoints	Cutpoints	Cutpoints
*Time period of pre-treatment outcome relative to treatment in months*	1	1	1	1	1	1
*Length of time period after treatment included in months*	13	24	36	13	24	36

I provide results for a Coarsened Exact Matching and subsequent difference-in-differences approach with *IVOL*_*i*,*m*_ as dependent variable

Relative matching time is the month before the treatment, i.e., the receipt of an ESG rating

The information provided for the matching variables shows whether coarsening was performed based on fixed cutpoints (“Cutpoints”) or automatically (“Automatic”) for each included variable

The symbols ****, ***, **, and * denote significance at the one per mill, five per mill, one percent, and five percent level, respectively

Coefficients with *p*-values ≥ 0.05 are not labeled as significant

**Table 5:** Difference-in-differences approach applied on IVOL of stocks receiving their first ESG rating (i.e., the treatment) and IVOL of stocks without ESG rating (control group) based on CE Matching by Fama-French-five-factor-loadings

	(1)	(2)	(3)	(4)	(5)	(6)
*Time period*	Jan 1999–June 2009	Jan 1999–June 2009	Jan 1999–June 2009	July 2009–Dec 2018	July 2009–Dec 2018	July 2009–Dec 2018
*Mean difference treated*	−0.25	−0.25	−0.37	−0.09	−0.43	−0.49
*Mean difference controls*	−0.04	−0.07	−0.22	−0.11	−0.10	0.01
*Conditional difference-in-differences (standard error of differences between treated and control group in parentheses)*	−0.20**** (0.04)	−0.17**** (0.04)	−0.16**** (0.04)	0.02 (0.13)	−0.33* (0.16)	−0.50* (0.23)
*Total treatment effect for the treated within two-way fixed effects model (robust standard error in parentheses)*	−11 (0.11)	−14 (0.13)	−0.00 (0.14)	−0.03 (0.16)	−0.44* (0.20)	−0.46* (0.24)
*N treated matched*	1095	947	840	483	362	273
*N controls matched*	453	364	304	501	408	318
*N treated unmatched*	565	535	495	74	59	44
*Matching variables included:*						
Mkt_Loading	Automatic	Automatic	Automatic	Automatic	Automatic	Automatic
SMB_Loading	Automatic	Automatic	Automatic	Automatic	Automatic	Automatic
HML_Loading	Automatic	Automatic	Automatic	Automatic	Automatic	Automatic
RMW_Loading	Automatic	Automatic	Automatic	Automatic	Automatic	Automatic
CMA_Loading	Automatic	Automatic	Automatic	Automatic	Automatic	Automatic
*Time period of pre-treatment outcome relative to treatment in months*	1	1	1	1	1	1
*Length of time period after treatment included in months*	13	24	36	13	24	36

I provide results for a Coarsened Exact Matching and subsequent difference-in-differences approach with *IVOL*_*i*,*m*_ as dependent variable

Relative matching time is the month before the treatment, i.e., the receipt of an ESG rating

The information provided for the matching variables shows whether coarsening was performed based on fixed cutpoints (“Cutpoints”) or automatically (“Automatic”) for each included variable

The symbols ****, ***, **, and * denote significance at the one per mill, five per mill, one percent, and five percent level, respectively

Coefficients with *p*-values ≥ 0.05 are not labeled as significant

I check whether the findings can be confirmed in other countries by analyzing Canadian stocks (results are not tabulated). ESG ratings are available for 14,428 of 23,169 company-month observations in the years 2013 to 2018. The ESG ratings have a mean (median) value of 0.73 (0.45) and range between −1.5 and 5.7. The mean IVOL_i,m_ of the full Canadian sample is 1.55% with a standard deviation of 1.27%. A random effects panel regression with IVOL_i,m_ as dependent and a dummy that indicates whether a stock is rated (*HasESGRating*) as independent variable yields a coefficient for the dummy of −0.11 percentage points, a robust standard error of 0.03, and a statistical significance at the one per mill level; supporting the findings on the US sample.

## Discussion

The results of the present study show a statistically significant influence of ESG ratings on IVOL. Whether the magnitude of the documented relation is large enough to be of economic significance has yet to be discussed.

When stocks receive an ESG rating, the magnitude of the negative effect on IVOL varies, depending on the methodology, between 0.62 (see Table 2, model (10)) and 0.14 (see Table 5, conditional difference-in-differences, see also event study in Fig. 2) percentage points. When considering that the mean IVOL of stocks with ESG rating is 0.85 percentage points lower than the IVOL of stocks with no ESG rating, a certain selection bias of the rating providers has to be considered. The selection of rated companies is based on index membership and market capitalization. Over time, the ESG ratings usually have been provided for those stocks listed in the MSCI KLD 400 Social Index, S&P 500, MSCI USA Index, MSCI USA IMI Index, as well as the 1000 largest US companies. By design of the employed asset pricing factors, stocks with lower market capitalization are more likely to have higher IVOL—and they more likely do not receive an ESG rating. The economic magnitude of the receipt of an ESG rating should, therefore, be closer to the 0.14 percentage points derived by the methods that focus on the longitudinal profile than to the 0.62 percentage points as derived by methods with an emphasis on the cross section. Compared to the mean IVOL of the full sample of 1.99% with a standard deviation of 1.92 percentage points, a reduction in IVOL of 0.14 percentage points does not initially seem economically meaningful. However, for listed companies the costs associated with receiving an ESG rating are negligible. Since studies on ESG disclosure find that better ESG transparency improves firm value and decreases capital costs (Cheng et al. 2014; Erragragui 2018; Ng and Rezae 2015; Yu et al. 2018; Ghoul et al. 2011), even when the disclosed information displays weak ESG performance (Eliwa et al. 2021), getting an ESG rating appears to resemble free lunch—although small—for listed companies and their equity investors. Therefore, the risk-reducing effect of an ESG rating is economically significant.

Although stocks subject to a negative screen show statistically significant lower idiosyncratic risk during recessions than comparable stocks with an ESG rating but without a negative screen, the magnitude of the difference is hardly economically significant. In recessions, the IVOL of stocks subject to a negative screen is 0.15 percentage points lower than the IVOL of stocks without a negative screen (see Table 2, model (10)). It is important to notice that only 29 months in the dataset actually cover periods of recessions. Corresponding to less than a tenth of the observation period. Consequently, the average risk reducing effect of sin stocks in times of recessions seems economically marginal over the entire observation period. Furthermore, stocks subject to a negative screen show slightly higher IVOL in times of economic growth. Hence, my findings are in line with Blitz and Fabozzi (2017) how do not find an outperformance of sin stocks on average.

Whether investors shall push companies to improve their ESG ratings in order to make use of the negative relation between the ESG rating score and IVOL is an issue worth discussing. Standardized coefficients in a simple OLS regression indicate that a one-standard-deviation-increase of the ESG rating score leads to a 0.03 to 0.04 percentage points decrease in IVOL. This is in line with the results in Table 1 (standard deviation of ESG ratings of 0.81) and Table 3, model (10) (coefficient of *ESGRating* of −0.01), according to which an one-standard-deviation-increase of the ESG rating leads to a 0.81 * 0.01 = 0.01 percentage points decrease in IVOL. This decrease does not seem economically meaningful compared to a mean IVOL of 1.63 (median: 1.32) with a standard deviation of 1.20 in the subsample of rated stocks. Also, when considering the typical IVOL of stocks in the portfolios formed by Stambaugh et al. (2015), the stocks with an ESG rating are unlikely to be in the highest-IVOL-portfolios. As the differences in the benchmark-adjusted returns of the two portfolios with the next lowest IVOL are rather negligible, it is unlikely that a decrease in IVOL triggered by a higher ESG rating has an economically meaningful impact on expected stock returns. It is important to notice that a better ESG performance—and consequently a higher ESG rating—might nevertheless have a positive influence on expected stock performance (for a review on this issue, see Liang and Renneboog 2020), however, unlikely via the influence of idiosyncratic risk on stock prices. On the flipside, the analysis provides no evidence that a better ESG rating might hurt investment performance.

## Conclusion

This paper contributes to the literature by providing empirical support on four important issues regarding the influence of ESG ratings on idiosyncratic stock risk. First, after the receipt of an ESG rating, idiosyncratic stock risk decreases. Second, stocks of rated companies show statistically and economically significantly lower idiosyncratic risk than stocks of companies with no ESG rating—even when stocks receive a low ESG rating. Third, stocks subject to a negative screen show statistically significant lower idiosyncratic risk during recessions than comparable stocks with an ESG rating but without a negative screen. Hence, as ESG ratings and negative screens individually influence stock risk, they should be considered separately. Fourth, the described effects are robust over time, statistically significant for US and Canadian stocks, and cannot be explained by exposure to liquidity risk, mispricing, innovations in volatility risk, investor sentiment, analysts’ forecast divergence and coverage, and firms’ age, size, and industry. Hence, the analysis shows that the previously found negative relation between stocks’ IVOL and ESG ratings is robust to the stocks’ exposure to liquidity risk, mispricing, innovations in volatility risk, investor sentiment, analysts’ forecast divergence and coverage, and firms’ age, size, and industry and also holds for sin stocks.

These findings have practical implications. The lower idiosyncratic risk of ESG-rated stocks—and of stocks with good ESG rating in particular—is not only good news for ESG investors or investors thinking about following an ESG investment approach, but also relevant for the remaining market participants. A lot of companies in developed and particularly in developing markets are not rated by an ESG rating agency yet. These companies should strive for receiving an ESG rating—even though the company might show a rather low ESG performance. Just the receipt of an ESG rating significantly reduces idiosyncratic stock risk. Admittedly, companies cannot compel rating providers to get a rating. However, ESG rating providers advertise with the number of covered companies and have an interest in increasing their coverage. Furthermore, the surprisingly large amount of historical rating changes (Berg et al. 2021; Drempetic et al. 2021) shows that many companies are hardly willing to collaborate or do not act thoroughly when they collaborate. Such behaviors can also prevent the receipt of a rating. Hence, investors should push companies to get rated and to act thoroughly and swiftly when contacted by rating providers.

Barber and Odean (2000, 2001), Polkovnichenko (2005), and Goetzmann and Kumar (2008) show that many investors (most probably not only ESG investors) are under-diversified and suffer from associated idiosyncratic risks. If investors are not willing to minimize their exposure to idiosyncratic risk by buying index funds (Oehler and Wanger 2020)^24^, they may have a better investment performance by investing in stocks of companies with high ESG ratings. However, although statistically significant in the cross sections of stocks, the economic magnitude of a higher ESG rating is rather small and may not justify the reallocation of an existing portfolio and the respective transaction costs (see Horn and Oehler 2020). But it may be worthwhile considering this effect when establishing a new portfolio from the scratch.

An advantage of the dataset employed in this study is the long history of ESG ratings. Yet, particularly since the financial crisis and especially in Europe, several rating agencies have become popular. Their rating approaches differ significantly from each other, sometimes leading to different assessments regarding the ESG performance of a company (Berg et al. 2022; Billio et al. 2020; Gibson et al. 2021). I do not assume that this has an influence on the results of this study, as MSCI/KLD had provided ESG ratings for US stocks even before some of the European rating agencies have been founded and therefore has the position of an old bull. Nevertheless, as differences in opinion (a proxy for uncertainty) significantly influence stocks’ IVOL (Diether et al. 2002; Anderson et al. 2009), further research might analyze the impact of ESG rating dispersion on IVOL. When doing so, researchers should consider ESG ratings and negative screens as independent from each other, as both have an individual influence on stock risk. Moreover, further research might expand the research questions of this paper to further stock markets, particularly since a lot of non-American firms have not received an ESG rating, yet.

It is beyond the scope of this paper to analyze the mechanisms that decrease idiosyncratic stock risk after the receipt of an ESG rating in detail. Nevertheless, it would be interesting to see further research on the question whether the reduction of information asymmetries and/or changes in institutional stock ownership drive the decrease in idiosyncratic stock risk.

## Footnotes

1 The percentage of stocks without an ESG rating varies considerably between the markets, e.g., developed markets have a higher rating coverage than developing markets. Larger companies are more likely to receive an ESG rating.

2 It is important to notice, though, that (some) ESG investors would keep investing solely in ESG assets even if these assets showed a financial underperformance (see Bauer et al. 2019). The reason is that ESG investments may additionally provide some non-financial utility (see Renneboog et al. 2008; El Ghoul and Karoui 2017). Furthermore, investments in ESG assets may have a lower systematic risk (see Albuquerque et al. 2019).

3 MSCI/KLD ESG ratings originally were provided by KLD. MSCI acquired KLD in 2010 and continues to provide the ratings with the original approach of KLD (MSCI/KLD ESG rating). The latter ratings are used in this analysis. MSCI additionally provides ESG ratings with a revised rating approach (https://www.msci.com/our-solutions/esg-investing/esg-ratings) but with a shorter history.

4 Other ESG rating providers like Refinitiv, ISS, Sustainalytics, and Vigeo Eiris started providing ESG ratings for US companies between 2002 and 2010. However, none of these providers reached a coverage comparable to MSCI/KLD until the year 2015.

5 See Hartzmark and Sussman (2019) for a comparable setting with ESG ratings for mutual funds.

6 In addition, Lins et al. (2017) find a negative correlation between their CSR measure and idiosyncratic risk for the period from August 2008 through March 2009. Lee and Faff (2009) find a negative relation between corporate social performance and idiosyncratic risk in the years 1998–2002, Luo and Bhattacharya (2009) in the years 2002 and 2003. Chen et al. (2018) analyze Taiwanese stocks from 2010–2014 and find a negative relation between CSR and idiosyncratic risk. In contrast, Cao et al. (2019) and Bofinger et al. (2020) show that the focus of ESG investors on ratings may lead to significant mispricing among high ESG-rated stocks, i.e., potentially higher idiosyncratic risk. Moreover, Becchetti et al. (2015) provide empirical support that stocks of companies with higher ESG ratings show higher idiosyncratic volatility. Glück et al. (2021) hardly find a significant impact of ESG rating changes on idiosyncratic volatility.

7 Ratings for most non-US stocks have been available only since 2013 (see MSCI 2016, p. 12 f.).

8 86,867 company-month observations are omitted from the original dataset because of the five US Dollar restriction. Furthermore, stocks with tickers C:SMU.UN, C:WRG, US:ACER, US:AMEH, US:APDN, US:ARWR, US:BLNK, US:ELOX, US:HROW, US:IDEX, US:IDRA, US:LFVN, US:LLEX, US:MAMS, US:OCAT, US:OTRK, US:PLX, US:PZZ, US:RIBT, US:SAUC, US:SRNE, US:SVRA, US:TEUM, US:TXMD, US:VTNR, US:XELB, and US:ZYXI are dropped because of data errors. The results stay robust when I exclude only stocks with a price lower than one US Dollar.

9 I abstain from additionally computing idiosyncratic risk with downside measures (lower partial moments, LPM) since Bouslah et al. (2018) report that results for LPM1 through LPM3 are similar to those for IVOL computed with the four-factor model.

10 http://mba.tuck.dartmouth.edu/pages/faculty/ken.french/data_library.html.

11 The factor data is from Robert Stambaugh’s homepage http://finance.wharton.upenn.edu/~stambaug/.

12 The factor data is from Robert Stambaugh’s homepage http://finance.wharton.upenn.edu/~stambaug/.

13 The factor data is from Jeffrey Wurgler’s homepage http://people.stern.nyu.edu/jwurgler/.

14 Stock price instead of earnings forecasts are used as companies’ profitability, and hence earnings, is already captured in the factor models. The applied standard deviation of analysts’ price forecasts and the number of analysts providing forecasts is from the Institutional Brokers Estimate System (I/B/E/S). The standard deviation of analysts’ price forecasts is divided by the stock price to adjust for price effects.

15 A vector of nine dummy variables is used to reflect firms’ industrial sector according to the MSCI Global Industry Classification Standard (GICS). The tenth sector *Communication* is omitted as basis vector because this is the sector with the fewest observations in the sample.

16 The data is from https://fred.stlouisfed.org/series/USREC.

17 According to Pástor and Veronesi (2003) the effect of firm age is very similar when age is measured as 1/(1+age), log(age), or even plain age.

18 This might also be the reason why mainstream investment organizations depict negative screening is the least beneficial approach for investment performance (see Amel-Zadeh and Serafeim 2018).

19 Please note, however, that the mispricing factors of Stambaugh and Yuan (2017) also include information captured in the factors RMW and CMA.

20 Tabulated detailed results of the respective regression analyses are available from the author upon request.

21 Please note that this might also lead to an undiversifiable risk in prices, however rather as a systematic risk (see Ben-David et al. 2018). It is an interesting question for further research whether the COVID-19 pandemic induced a further large preference shift towards ESG investments. The data currently at hand, unfortunately, does not enable such an analysis. Furthermore, it still needs to be analyzed whether a systematic COVID-19 factor exists to correctly disentangle systematic and idiosyncratic risks during the pandemic.

22 Tabulated detailed results of the respective regression analyses are available from the author upon request.

23 If the event window for a company would cover parts of both observation periods, e.g., when the first ESG rating of a company was received five months before the end of the financial crisis, the company is excluded from the difference-in-differences analysis.

24 Retail investors with ESG preferences even have the possibility to select a robo-advisor that manages their investments in ESG index funds automatically (see Horn et al. 2020).

## References

[CR1] Adler T, Kritzman M (2008). The cost of socially responsible investing. Journal of Portfolio Management.

[CR2] Albuquerque R, Koskinen Y, Zhang C (2019). Corporate social responsibility and firm risk: theory and empirical evidence. Management Science.

[CR3] Amel-Zadeh A, Serafeim G (2018). Why and how investors use ESG information: Evidence from a global survey. Financial Analysts Journal.

[CR4] Anderson E, Ghysels E, Juergens J (2009). The impact of risk and uncertainty on expected returns. Journal of Financial Economics.

[CR5] Ang A, Hodrick RJ, Xing Y, Zhang X (2006). The cross-section of volatility and expected returns. Journal of Finance.

[CR6] Ang A, Hodrick RJ, Xing Y, Zhang X (2009). High idiosyncratic risk and low returns: International and further US evidence. Journal of Financial Economics.

[CR7] Baker M, Wurgler J (2006). Investor sentiment and the cross-section of stock returns. Journal of Finance.

[CR8] Baker A, Larcker D, Wang C (2022). How much should we trust staggered difference-in-differences estimates?. Journal of Financial Economics.

[CR9] Bali T, Zhou H (2016). Risk, uncertainty, and expected returns. Journal of Financial and Quantitative Analysis.

[CR10] Barber BM, Odean T (2000). Trading is hazardous to your wealth: The common stock investment performance of individual investors. Journal of Finance.

[CR11] Barber BM, Odean T (2001). Boys will be boys: Gender, overconfidence, and common stock investment. Quarterly Journal of Economics.

[CR12] Barnett ML, Salomon RM (2006). Beyond dichotomy: The curvilinear relationship between social responsibility and financial performance. Strategic Management Journal.

[CR13] Bartram, S., G. Brown, and R. Stulz. 2016. Why does idiosyncratic risk increase with market risk? NBER Working Paper 22492. https://www.nber.org/papers/w22492. Accessed 9 January 2023.

[CR14] Bauer R, Ruof T, Smeets P (2019). Get real! Individuals prefer more sustainable investments. Working paper.

[CR15] Becchetti L, Ciciretti R, Giovanelli A (2013). Corporate social responsibility and earnings forecasting unbiasedness. Journal of Banking and Finance.

[CR16] Becchetti L, Ciciretti R, Hasan I (2015). Corporate social responsibility, stakeholder risk, and idiosyncratic volatility. Journal of Corporate Finance.

[CR17] Ben-David I, Franzoni F, Moussawi R (2018). Do ETFs increase volatility?. Journal of Finance.

[CR18] Benson KL, Humphrey JE (2008). Socially responsible investment funds: Investor reaction to current and past returns. Journal of Banking & Finance.

[CR19] Berg F, Fabisik K, Sautner Z (2021). Is history repeating itself? The (un)predictable past of ESG ratings.

[CR20] Berg F, Koelbel J, Rigobon R (2022). Aggregate confusion: the divergence of ESG ratings. Review of Finance.

[CR21] Bialkowski J, Starks LT (2016). SRI funds: Investor demand, exogenous shocks, and ESG profiles.

[CR22] Billio M, Costola M, Hristova I, Latino C, Pelizzon L (2020). Inside the ESG ratings: (Dis)agreement and performance. Working paper.

[CR23] Blackwell M, Iacus S, King G, Porro G (2009). cem: Coarsened exact matching in Stata. Stata Journal.

[CR24] Blitz D, Fabozzi F (2017). Sin stocks revisited: resolving the sin stock anomaly. Journal of Portfolio Management Fall.

[CR25] Boehme RD, Danielsen BR, Kumar P, Sorescu SM (2009). Idiosyncratic risk and the cross-section of stock returns: Merton (1987) meets Miller (1977). Journal of Financial Markets.

[CR26] Boehmer E, Kelley E (2009). Institutional investors and the informational efficiency of prices. Review of Financial Studies.

[CR27] Bofinger Y, Heyden KJ, Rock B (2020). Corporate social responsibility and market efficiency: evidence from ESG and misvaluation measures. Working paper.

[CR28] Bouslah K, Kryzanowski L, M’Zali B (2013). The impact of the dimensions of social performance on firm risk. Journal of Banking & Finance.

[CR29] Bouslah K, Kryzanowski L, M’Zali B (2018). Social performance and firm risk: impact of the financial crisis. Journal of Business Ethics.

[CR30] Bozhkov S, Lee H, Sivarajah U, Despoudi S, Nandy M (2020). Idiosyncratic risk and the cross-section of stock returns: the role of mean-reverting idiosyncratic volatility. Annals of Operations Research.

[CR31] Brenner M, Izhakian Y (2018). Asset pricing and ambiguity: Empirical evidence. Journal of Financial Economics.

[CR33] Cao J, Han B (2016). Idiosyncratic risk, costly arbitrage, and the cross-section of stock returns. Journal of Banking & Finance.

[CR32] Cao C, Simin T, Zhao J (2008). Can growth options explain the trend in idiosyncratic risk?. Review of Financial Studies.

[CR34] Cao JJ, Titman S, Zhan XE, Zhang WE (2019). ESG preference and market efficiency: Evidence from mispricing and institutional trading. Working paper.

[CR35] Carhart MM (1997). On persistence in mutual fund performance. Journal of Finance.

[CR36] Chava S (2014). Environmental externalities and cost of capital. Management Science.

[CR37] Chen RCY, Hung S-W, Lee C-H (2018). Corporate social responsibility and firm idiosyncratic risk in different market states. Corporate Social Responsibility and Environmental Management.

[CR38] Cheng B, Ioannou I, Serafeim G (2014). Corporate social responsibility and access to finance. Strategic Management Journal.

[CR39] Chichernea D, Ferguson M, Kassa H (2015). Idiosyncratic risk, investor base, and returns. Financial Management.

[CR40] Cooper E, Uzun H (2019). Corporate social responsibility and bankruptcy. Studies in Economics and Finance.

[CR41] Dettmann, E., A. Giebler, and A. Weyh. 2020. flexpaneldid a Stata toolbox for causal analysis with varying treatment time and duration. https://papers.ssrn.com/sol3/papers.cfm?abstract_id=3692458. Accessed 9 January 2023.

[CR42] Diether KB, Malloy CJ, Scherbina A (2002). Differences of opinion and the cross section of stock returns. Journal of Finance.

[CR44] Drempetic S, Klein C, Zwergel B (2020). The influence of firm size on the ESG score: corporate sustainability ratings under review. Journal of Business Ethics.

[CR43] Drempetic S, Hoepner A, Klein C (2021). Change my ESG history: Ex-post deletions and additions. 3rd Conference on Behavioral Research in Finance, Governance, and Accounting, virtual, November 4–5, 2021.

[CR45] Dunn J, Fitzgibbons S, Pomorski L (2018). Assessing risk through environmental, social and governance exposures. Journal of Investment Management.

[CR46] Duppati G, Kijkasiwat P, Hunjra AI, Liew CY (2022). Do institutional ownership and innovation influence idiosyncratic risk?. Global Finance Journal.

[CR47] El Ghoul S, Karoui A (2017). Does corporate social responsibility affect mutual fund performance and flows?. Journal of Banking & Finance.

[CR48] Eliwa Y, Aboud A, Saleh A (2021). ESG practices and the cost of debt: Evidence from EU countries. Critical Perspectives on Accounting.

[CR49] Erragragui E (2018). Do creditors price firms’ environmental, social and governance risks?. Research in International Business and Finance.

[CR50] Fabozzi F, Ma KC, Oliphant B (2008). Sin stock returns. Journal of Portfolio Management.

[CR51] Fama EF, French KR (1992). The cross-section of expected stock returns. Journal of Finance.

[CR52] Fama EF, French KR (2015). A five-factor asset pricing model. Journal of Financial Economics.

[CR53] Ferreira M, Laux P (2007). Corporate governance, idiosyncratic risk, and information flow. Journal of Finance.

[CR54] Friede G, Busch T, Bassen A (2015). ESG and financial performance: aggregated evidence from more than 2000 empirical studies. Journal of Sustainable Finance & Investment.

[CR55] Geczy C, Stambaugh R, Levin D (2003). Investing in socially responsible mutual funds. Working paper.

[CR56] Ghoul S, Guedhami O, Kwok C, Mishra D (2011). Does corporate social responsibility affect the cost of capital?. Journal of Banking & Finance.

[CR57] Gibson R, Krueger P, Schmidt P (2021). ESG rating disagreement and stock returns. Financial Analysts Journal.

[CR58] Giese G, Lee L-E, Melas D, Nagy Z, Nishikawa L (2019). Foundations of ESG investing: How ESG affects equity valuation, risk, and performance. Journal of Portfolio Management.

[CR59] Glück, M., B. Hübel, and H. Scholz. 2021. ESG rating events and stock market reactions. https://papers.ssrn.com/sol3/papers.cfm?abstract_id=3803254. Accessed 9 January 2023.

[CR60] Goetzmann WN, Kumar A (2008). Equity portfolio diversification. Review of Finance.

[CR61] Goss A, Roberts G (2011). The impact of corporate social responsibility on the cost of bank loans. Journal of Banking & Finance.

[CR62] Hartzmark SM, Sussman AB (2019). Do investors value sustainability? A natural experiment examining ranking and fund flows. Journal of Finance.

[CR63] Hong H, Kacperczyk M (2009). The price of sin: The effects of social norms on markets. Journal of Financial Economics.

[CR64] Horn M, Oehler A (2020). Automated portfolio rebalancing: Automatic erosion of investment performance?. Journal of Asset Management.

[CR65] Horn M, Oehler A, Wendt S, Walker T, Gramlich D, Bitar M, Fardnia P (2020). FinTech for consumers and retail investors: opportunities and risks of digital payment and investment services. Ecological, societal, and technological risks and the financial sector.

[CR66] Killins R, Ngo T, Wang H (2020). The underpricing of sin stocks. Journal of Investing.

[CR67] Kim M, Surroca J, Tribó J (2014). Impact of ethical behavior on syndicated loan rates. Journal of Banking & Finance.

[CR68] King G, Nielsen R (2019). Why propensity scores should not be used for matching. Political Analysis.

[CR69] Krueger P (2015). Corporate goodness and shareholder wealth. Journal of Financial Economics.

[CR70] Latino, C., L. Pelizzon, and A. Rzeznik. 2021. The power of ESG ratings on stock markets. https://papers.ssrn.com/sol3/papers.cfm?abstract_id=3801703. Accessed 9 January 2023.

[CR71] Lee DD, Faff RW (2009). Corporate sustainability performance and idiosyncratic risk: a global perspective. Financial Review.

[CR72] Levy H (1978). Equilibrium in an imperfect market: A constraint on the number of securities in the portfolio. American Economic Review.

[CR73] Liang H, Renneboog L (2020). Corporate social responsibility and sustainable finance: a review of the literature.

[CR74] Lins KV, Servaes H, Tamayo A (2017). Social capital, trust, and firm performance: The value of corporate social responsibility during the financial crisis. Journal of Finance.

[CR75] Lintner J (1965). The valuation of risk assets and the selection of risky investments in stock portfolios and capital budgets. The Review of Economics and Statistics.

[CR76] Luo X, Bhattacharya CB (2009). The debate over doing good: corporate social performance, strategic marketing levers, and firm-idiosyncratic risk. Journal of Marketing.

[CR77] Mishra S, Modi SB (2013). Positive and negative corporate social responsibility, financial leverage, and idiosyncratic risk. Journal of Business Ethics.

[CR78] Monti, A., P. Pattitoni, B. Petracci, and O. Randl. 2019. Does corporate social responsibility impact equity risk? International evidence. https://papers.ssrn.com/sol3/papers.cfm?abstract_id=3167883. Accessed 9 January 2023.

[CR79] Moskowitz TJ, Grinblatt M (1999). Do industries explain momentum?. Journal of Finance.

[CR80] Mossin J (1966). Equilibrium in a capital market. Econometrica.

[CR81] MSCI (2016). MSCI ESG KLD STATS: 1991–2015 data sets.

[CR82] Nandy M, Lodh S (2012). Do banks value the eco-friendliness of firms in their corporate lending decision? Some empirical evidence. International Review of Financial Analysis.

[CR83] Ng A, Rezae Z (2015). Business sustainability performance and cost of equity capital. Journal of Corporate Finance.

[CR84] Oehler, A. 2013. Minimum standards for ecologically and socially responsible investments. https://www.uni-bamberg.de/fileadmin/uni/fakultaeten/sowi_lehrstuehle/finanzwirtschaft/Transfer/Anforderungen_Mindeststandard_fuer_sozial_oekologische_Geldanlagen_final_Dec_2013.pdf. Accessed 9 January 2023.

[CR86] Oehler A, Schneider J (2022). Gambling with lottery stocks?. Journal of Asset Management.

[CR87] Oehler A, Wanger HP (2020). Household portfolio optimization with XTFs? An empirical study using the SHS-base. Research in International Business and Finance.

[CR85] Oehler A, Horn M, Wendt S, Walker T, Kibsey SD, Crichton R (2018). Why self-commitment is not enough: On a regulated minimum standard for ecologically and socially responsible financial products and services. Designing a sustainable financial system: development goals and Socio-ecological responsibility.

[CR88] Pástor L, Stambaugh RF (2003). Liquidity risk and expected stock returns. Journal of Political Economy.

[CR89] Pástor L, Stambaugh RF (2019). Liquidity risk after 20 years.

[CR90] Pástor L, Veronesi P (2003). Stock valuation and learning about profitability. Journal of Finance.

[CR91] Polkovnichenko V (2005). Household portfolio diversification: A case for rank-dependent preferences. Review of Financial Studies.

[CR92] Renneboog L, Ter Horst J, Zhang C (2008). Socially responsible investments: Institutional aspects, performance, and investor behavior. Journal of Banking & Finance.

[CR93] Richey G (2020). Is it good to sin when times are bad? An investigation of the defensive nature of sin stocks. Journal of Investing.

[CR94] Riedl A, Smeets P (2017). Why do investors hold socially responsible mutual funds?. Journal of Finance.

[CR95] Ryan A, Kontopantelis E, Linden A, Burgess J (2018). Now trending: Coping with non-parallel trends in difference-in-differences analysis. Statistical Methods in Medical Research.

[CR96] Sassen R, Hinze A-K, Hardeck I (2016). Impact of ESG factors on firm risk in Europe. Journal of Business Economics.

[CR97] Serafeim, G., and A. Yoon. 2021. Stock price reactions to ESG news: the role of ESG ratings and disagreement. https://papers.ssrn.com/sol3/papers.cfm?abstract_id=3765217. Accessed 9 January 2023.

[CR98] Sharpe WF (1964). Capital asset prices: A theory of market equilibrium under conditions of risk. Journal of Finance.

[CR99] Social Investment Forum (SIF). 2018. Report on US sustainable, responsible and impact investing trends 2018. https://www.ussif.org/files/Trends/Trends%202018%20executive%20summary%20FINAL.pdf. Accessed 9 January 2023.

[CR102] Stambaugh RF, Yuan Y (2017). Mispricing factors. Review of Financial Studies.

[CR100] Stambaugh RF, Yu J, Yuan Y (2012). The short of it: Investor sentiment and anomalies. Journal of Financial Economics.

[CR101] Stambaugh RF, Yu J, Yuan Y (2015). Arbitrage asymmetry and the idiosyncratic volatility puzzle. Journal of Finance.

[CR103] Statman M (2000). Socially responsible mutual funds. Financial Analysts Journal.

[CR104] Welch BL (1947). The generalization of ‘student’s’ problem when several different population variances are involved. Biometrika.

[CR105] Wong W, Batten J, Ahmad A, Mohamed-Arshad A, Nordin S, Adzis A (2021). Does ESG certification add firm value?. Finance Research Letters.

[CR106] Yu E, Guo C, Luu B (2018). Environmental, social and governance transparency and firm value. Business Strategy and the Environment.

[CR107] Zerbib, O. 2020. A sustainable capital asset pricing model (S-CAPM): Evidence from green investing and sin stock exclusion. https://papers.ssrn.com/sol3/papers.cfm?abstract_id=3455090. Accessed 9 January 2023.

